# The Floating Acromion: A Rare and Previously Unreported Injury Possibly Requiring Surgical Stabilization

**DOI:** 10.1155/2020/9465370

**Published:** 2020-07-15

**Authors:** Simone J. M. Stoots, Robert J. Derksen

**Affiliations:** Department of Trauma Surgery, Zaandam Medical Center, Kon. Julianaplein 58, 1502 DV Zaandam, Netherlands

## Abstract

Acromion fractures are increasingly seen as a postoperative complication following reversed shoulder arthroplasty. However, traumatic fractures of the acromion, usually caused by direct trauma, are rare. Therefore, the current literature lacks standardized clinical guidelines regarding the surgical treatment of these kinds of fractures. We present a traumatic acromion fracture and concomitant distal clavicle fracture, resulting in a so-called “floating acromion.” A fifty-four-year-old female patient was presented at the Emergency Department following a fall from the stairs. She complained of severe pain in the left shoulder. Radiographic evaluation of the left shoulder revealed an acromion fracture and concomitant distal clavicle fracture. Initially, since there was no dislocation, this “floating acromion” was treated conservatively. However, after 4 weeks, no improvement in pain was seen and a control CT scan revealed no callus formation. Considering the possibility that this could be a biomechanically unstable injury, together with the persistent severe pain, it was decided to proceed with surgical treatment. A lateral clavicle plate was used to stabilize the acromion fracture. Postoperatively, the patient was provided with a sling. She was regularly seen at the outpatient clinic. After two weeks of circumduction exercises, she was allowed to build up active movement under the supervision of a shoulder physiotherapist. Nevertheless, she developed a frozen shoulder. However, our patient fully recovered with complete restoration of shoulder function. Therefore, for operative management of acromion fractures, we suggest the use of a lateral clavicle plate which fits remarkably well on the lateral spine and acromion.

## 1. Introduction

The acromion, or acromial process, is the most lateral bony projection of the scapula and forms the outer angle of the shoulder. Fractures of the acromion are rare and represent approximately 6-12% of scapular fractures [1] which, in turn, represent only 0,3% of all fractures [2]. Although traumatic acromion fractures, which are usually caused by direct trauma on the shoulder, occur infrequently, several cases have been published associating acromial base fractures with reversed shoulder arthroplasty, considering it a postoperative complication [3]. We present a rare case of a traumatic acromion fracture and concomitant distal clavicle fracture resulting in a so-called “floating acromion.”

## 2. Case Presentation

A fifty-four-year-old, right-hand dominant, healthy female patient was presented at the Emergency Department following a fall from the stairs. She complained of severe pain in her left shoulder and in the back. Besides ecchymosis and prominent swelling, an inspection of the left shoulder did not show any abnormalities. There were no neurovascular deficits. On palpation, there was marked tenderness of the acromion.

**Figure 1:**
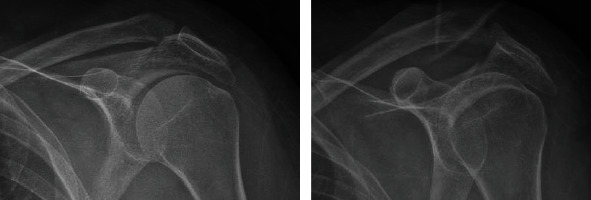
Anteroposterior X-ray imaging showing an acromion fracture and concomitant distal clavicle fracture.

**Figure 2:**
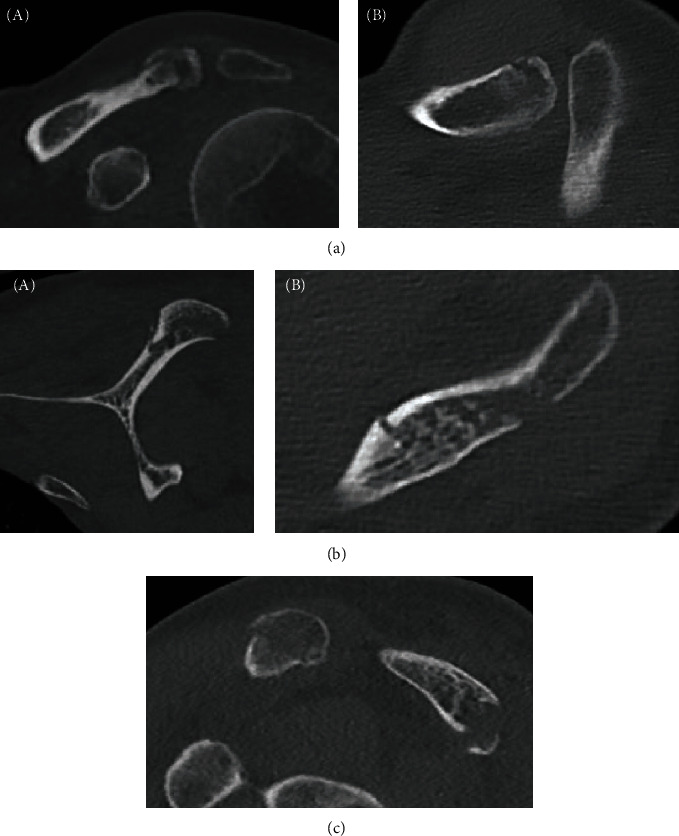
(a) Coronal (A) and transversal (B) view of the CT scan showing the distal clavicle fracture. (b) Coronal (A) and transversal (B) view of the CT scan showing the acromion fracture. (c) Sagittal view of the CT scan showing the acromion fracture and concomitant distal clavicle fracture.

Radiographic evaluation of the left shoulder by both conventional radiographic imaging and additional computed tomography (CT) scan revealed a distal clavicle fracture accompanied by a complete fracture of the acromion, Type 1B according to Kuhn's classification system [4], with a maximum fracture gap of five millimeters (Figures 1 and 2). The combination of these two fractures gives rise to an entity that, to the best of our knowledge, has not been described in the literature before: the “floating acromion.”

Initially, the patient was given a Gilchrist shoulder immobilizer for one week followed by an arm sling for another three weeks in terms of conservative treatment. However, four weeks after her injury, the patient still suffered from severe pain in the left shoulder which had not improved at all. Repeated radiographic imaging showed neither further dislocation nor callus formation of both the acromion and distal clavicle fracture. Also, signs of bone formation were absent. As this floating acromion was considered to possibly be a biomechanically unstable injury, it was decided to proceed with surgical treatment.

**Figure 3:**
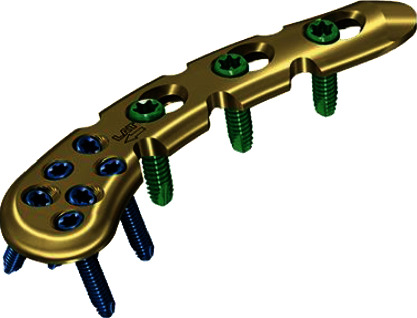
Lateral clavicle plate (Synthes).

**Figure 4:**
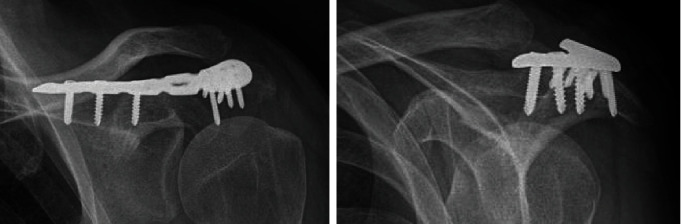
X-ray imaging two weeks postsurgery showing the usage of a lateral clavicle plate as a bridging plate for an acromion fracture.

**Figure 5:**
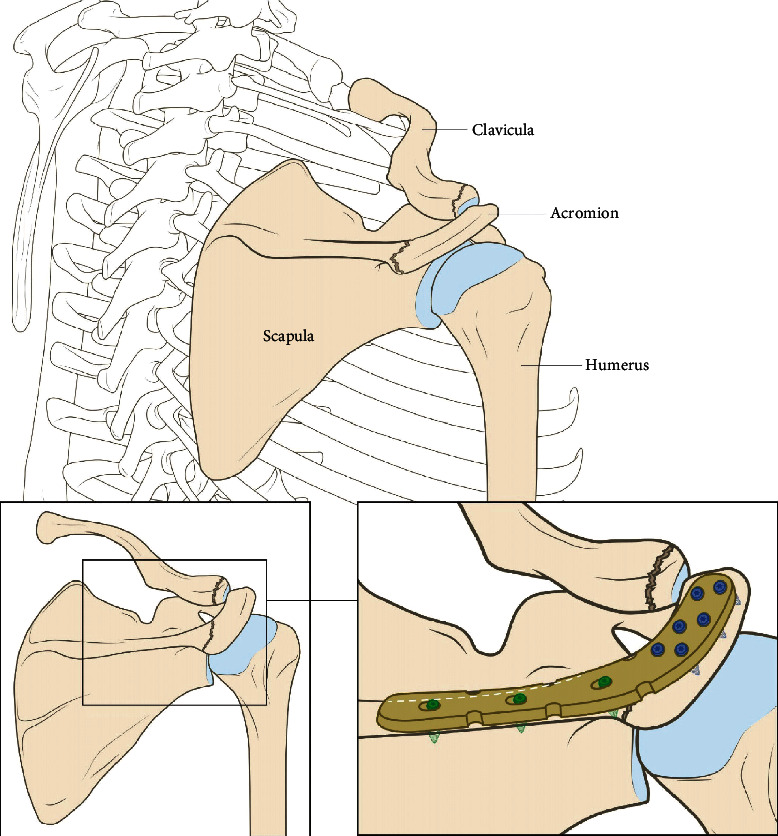
Illustration of a lateral clavicle plate used as a bridging plate for an acromion fracture.

In September 2017, an open reduction and internal fixation (ORIF) of the acromion fracture was performed. The patient was positioned in a beach chair on a shoulder modular table of which the ipsilateral shoulder part was removed for optimal access to the acromion and to be able to perform the procedure under fluoroscopic control. An incision was made over the lateral part of the scapular spine and extended over the dorsal acromion. The supraspinatus muscle was elevated from the lateral scapular spine. On the distal side of the fracture, the acromion was cleared from deltoid fibers to accommodate the positioning of the plate. During surgery, it was confirmed that there were no signs of callus formation, as preoperative radiographic imaging already suggested. After revitalizing the inert fracture edges, a lateral clavicle plate (Synthes, Figure 3) was used as a bridging plate (Figures 4 and 5). The plate was not contoured before fixation. Six bicortical minifragment (2,7 mm) angular stable screws were put in the acromion and three bicortical small fragment (3,5 mm) screws (two cortical screws and one angular stable screw) in the lateral scapular spine. After replacing the supraspinatus back over the plate, the wound was closed with noninterrupted absorbable sutures. As the direct postoperative period was uneventful, the patient was discharged from the hospital one day postsurgery. She was provided with a sling and instructed to do circumduction exercises in the first two weeks. After that, four weeks of active movement was allowed up to ninety degrees in abduction and anteflexion.

Postsurgery, the patient was regularly seen at the outpatient clinic. The wound healed without complications. In order to regain full range of motion, the patient visited a shoulder physiotherapist regularly. Six weeks postsurgery, she had already noted a significant improvement in pain, scored by the visual analogue scale, as well as in functional outcome regarding the range of motion compared to the initial conservative treatment. However, as some deep shoulder pain persisted, an ultrasound was performed to assess possible concomitant rotator cuff injuries, which were not seen. Presumably, she had developed a frozen shoulder and she fully recovered with complete restoration of shoulder function.

For anatomical purposes, the lateral clavicle plate that was used to fixate the acromion could only be placed superficially under the skin. As over time the patient experienced discomfort from the plate, plate removal was performed after complete fracture healing was confirmed by CT imaging.

## 3. Discussion

Although traditionally nondisplaced acromion fractures are treated conservatively, nonoperative treatment does carry a risk of symptomatic acromion nonunion development [5]. Therefore, over the last twenty years, especially in working adults, ORIF is increasingly suggested [5]. Other indications for a surgical approach include displaced or open fractures and multiple disruptions of the superior shoulder suspensory complex [6].

Although the current literature lacks established clinical guidelines or standardized algorithms regarding surgical treatment of acromion fractures, several suggestions have been made. Nasab, for example, suggested open reduction and a pin fixation of an isolated acromion fracture [7], and Weber described a tension band wiring of a displaced fracture of the posterior angle of the acromion [8].

In floating shoulder injuries, defined as ipsilateral fractures of the clavicle and the scapular neck, surgical treatment usually consists of ORIF of the clavicle [9]. Stabilizing the clavicle not only remodels the shoulder, it also indirectly minimizes concomitant dislocated scapular fractures and improves functional outcome compared to conservative treatment alone [10, 11]. As the injuries involved in this case are biomechanically comparable to those in floating shoulder injuries (both the anterior and posterior stabilizing columns are disrupted), the initial thought was to likewise stabilize the distal clavicle fracture.

However, due to the fracture characteristics in this instance like the extremely lateral fracture line of the clavicle, a lateral clavicle plate would be suitable to stabilize the clavicle. Obviously, an acromion hook plate would also not have provided a stable situation, since the necessary counterforce was lacking to uphold the acromion. Hence, to achieve optimal stabilization, we chose to stabilize the acromion fracture instead by using a lateral clavicle plate and placing it across the scapular spine over the acromion. In the existing literature, we could not find any report of the lateral clavicle plate being used for the open reduction and internal fixation of an acromion fracture. In this case, and on a prior case of an isolated acromion fracture nonunion, we found that the plate fits excellently on the lateral spine and acromion. Using this surgical technique, complete shoulder function and range of motion were achieved in this instance.

## 4. Conclusion

Fractures of the acromion are rare injuries, and as the current literature lacks standardized clinical guidelines regarding surgical treatment, they can be challenging to manage. In this case of a traumatic complete posterior acromion fracture combined with a distal clavicle fracture, an unstable acromial complex had developed. Hence, we are introducing a new term within trauma and shoulder surgery: the floating acromion. For operative management of this rare injury, we suggest the use of a lateral clavicle plate on the lateral spine and acromion. Although normally not indicated for this type of injury, the lateral clavicle plate fits perfectly on the scapular spine and acromion and can therefore be considered as a suitable alternative to stabilize the acromion.

## References

[1] Court-Brown C., McQueen M., Tornetta P. (2006). *Trauma – Orthopaedic Surgery Essentials*.

[2] Court-Brown C. M., Caesar B. (2006). Epidemiology of adult fractures: a review. *Injury*.

[3] Trevor C. (2011). Acromial base fractures after reverse total shoulder arthroplasty: report of five cases. *Journal of Shoulder and Elbow Surgery*.

[4] Kuhn J. E., Blasier R. B., Carpenter J. E. (1994). Fractures of the acromion process: a proposed classification system. *Journal of Orthopaedic Trauma*.

[5] Hess F., Zettl R., Welter J., Smolen D., Knoth C. (2019). The traumatic acromion fracture: review of the literature, clinical examples and proposal of a treatment algorithm. *Archives of Orthopaedic and Trauma Surgery*.

[6] Hill B. W., Anavian J., Jacobson A. R., Cole P. A. (2014). Surgical management of isolated acromion fractures: technical tricks and clinical experience. *Journal of Orthopaedic Trauma*.

[7] Nasab S. A. M. (2013). Isolated displaced fracture of the acromion: a rare case report and the consequence of treatment by open reduction and pin fixation. *Archives of Trauma Research*.

[8] Weber D., Sadri H., Hoffmeyer P. (2000). Isolated fracture of the posterior angle of the acromion: a case report. *Journal of Shoulder and Elbow Surgery*.

[9] Bartoníček J., Tuček M., Naňka O. (2018). Floating Shoulder: Myths and Reality. *JBJS Reviews*.

[10] DeFranco M. J., Patterson B. M. (2010). The floating shoulder Redefined. *The Journal of the American Academy of Orthopaedic Surgeons*.

[11] Yadav V., Khare G. N., Singh S. (2013). A prospective study comparing conservative with operative treatment in patients with a ‘floating shoulder’ including assessment of the prognostic value of the glenopolar angle. *The Bone & Joint Journal*.

